# Mechanism Research on Thieno-Fused Bis-BODIPY Bifunctional Photosensitizers

**DOI:** 10.3390/ma19101987

**Published:** 2026-05-11

**Authors:** Yuejia Wang, Di Wang, Xinyu Chen, Yishan Sun, Guoguo Shi, Jianfang Cao

**Affiliations:** 1 School of Chemical Engineering, Ocean and Life Sciences, Panjin Campus, Dalian University of Technology, Panjin 124221, China; 2339120564@mail.dlut.edu.cn (Y.W.);; 2 Leicester International Institute, Panjin Campus, Dalian University of Technology, Panjin 124221, China

**Keywords:** photodynamic therapy, thieno-fused BODIPY, donor engineering, mechanical explanation, intersystem crossing

## Abstract

This study is dedicated to the development of novel boron-dipyrromethene (BODIPY)-based photosensitizers, focusing on investigating the regulatory mechanism of introducing different electron-donating groups at the α-position on the photosensitizing performance of thieno-bis-BODIPY derivatives, aiming to provide a theoretical basis for cancer photodynamic therapy (PDT). Five thieno-bis-BODIPY molecules (FD1-FD5) were constructed by connecting two BODIPY units via a thiophene π-bridge and introducing various substituents at the α-position of their phenyl groups. Systematic theoretical studies revealed that unilaterally substituted molecules exhibit superior photophysical properties compared to their bilaterally substituted counterparts. The key mechanisms involve structural planarization, increased electrostatic potential difference, and the formation of hybrid LE/CT characteristics in the excited state, all of which collectively promote the intersystem crossing (ISC) process. Specifically, the pyrrole-substituted FD4 exhibits the highest ISC efficiency due to its stronger electron-donating ability and greater molecular planarity, and it is predicted to possess a stronger singlet oxygen generation capability than the methoxy-substituted FD2 while maintaining fluorescence emission. In contrast, bilateral substitution leads to structural distortion, which favors fluorescence emission, as seen in FD5 which exhibits the longest absorption wavelength. This research elucidates the key mechanisms for enhancing ISC and photosensitizing performance from the perspectives of electronic structure and excited-state characteristics, providing theoretical guidance for overcoming limitations such as insufficient tissue penetration in traditional BODIPY photosensitizers and clarifying structure–activity relationships.

## 1. Introduction

The global burden of cancer is increasing, while traditional therapies face many limitations due to their strong toxic side effects and poor selectivity [1]. Photodynamic therapy (PDT), owing to its combination of therapy and minimally invasive nature, has become a highly regarded approach in cancer treatment [2,3]. However, the structure–property relationship of photosensitizers remains unclear, and conventional photosensitizers often suffer from issues such as insufficient tissue penetration depth and low treatment specificity, which constrain the development of PDT [4,5]. Modified boron-dipyrromethene (BODIPY) dyes, as an excellent class of photosensitizers, can selectively accumulate in cancer cells and exhibit good tumor affinity, thus attracting considerable attention [6,7,8]. PDT destroys tumor cells through photophysical energy level transitions of the photosensitizer and subsequent photochemical processes mediated by Type I (radical-mediated) and Type II (singlet oxygen-mediated) mechanisms, in which properties of the photosensitizer such as excited-state lifetime and singlet oxygen quantum yield directly affect therapeutic efficacy [8,9,10]. Designing highly efficient Type II photosensitizers requires meeting several key criteria: long-wavelength light-harvesting capability to enhance tissue penetration and light energy utilization; high triplet-state population efficiency, which involves optimizing the intersystem crossing quantum yield to ensure sufficient T_1_-state population and the T_1_-state energy must be higher than the ground-state energy level of triplet oxygen (>0.980 eV) to satisfy the energy transfer threshold [11].

In recent years, the strategy of constructing BODIPY derivatives containing electron donors and acceptors to enhance the ISC ability has gradually attracted the attention of scientists. The first step of improvement took place in effectiveness. The research group led by Li Jiazhu has developed a series of asymmetric benzothiophene-fused BODIPY dyes which shows that the dyes can simultaneously generate singlet oxygen and superoxide radicals with high ROS generation efficiency while confirming its good biocompatibility and low dark toxicity [12]. Professor Zhao Jianzhang found that conjugating a thiophene ring with BODIPY can significantly redshift its maximum absorption peak, enabling the extension in absorption wavelength of BODIPY and confirmed the competitive mechanism between fluorescence emission and ^1^O_2_ generation [13]. The second breakthrough was in the NIR-II window and exploration of preclinical translation. In 2025, Gong’s group synthesized fully conjugated BODIPY with intense NIR absorption and excellent photophysical properties which concludes that precise regulation of singlet-triplet energy levels enables application in next-generation materials [14]. Simultaneously, Song’s group reported that de novo designed BODIPY oligomers with excellent NIR-II optical properties suppressed aggregation and enhanced stability and can effectively overcome the poor blood circulation half-life of BODIPY materials when delivered via interventional methods [15].

**Figure 1:**
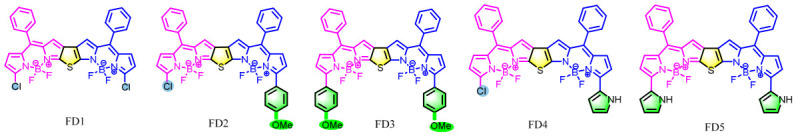
Schematic diagram of the molecular structure of FD1-5. (Pink represents BODIPY 1, yellow represents the thiophene ring, blue represents BODIPY 2, and green represents the α-position linked donor group).

Research group of Jiao has synthesized a series of π-conjugation-extended thieno-bis-BODIPY molecules, where two BODIPY units are linked via a thiophene π-bridge. Each BODIPY unit is attached to a phenyl group at the meso position and different active substituents at the α-positions on both sides, resulting in five thieno-bis-BODIPY molecules (FD1–FD5) with distinct properties, as Figure 1 [16]. Although experiments have confirmed that all five molecules exhibit strong fluorescence emission capability [16], whether they possess the ability to generate ROS and the specific effects of substitution patterns (monosubstitution vs. disubstitution) on the excited-state dynamics of sulfur-fused dimers remain unclear. Through systematic theoretical comparison, this study reveals for the first time from a mechanistic perspective that monosubstitution induces significant molecular planarization and enhances the electrostatic potential gradient, thereby driving intramolecular electron redistribution and transfer, increasing the proportion of charge-transfer (CT) states, and activating efficient ISC channels; in monosubstituted systems, especially those with a strong pyrrole donor, the S_2_ → T_3_ ISC pathway becomes dominant; the mixing of LE/CT character in the S_2_ state together with the CT character in the T_3_ state constitutes the key to high ISC efficiency. Based on these findings, the monosubstituted system (FD4) is predicted to possess the ability to generate ^1^O_2_. These discoveries not only extend existing concepts but also establish structure–mechanism–property relationships for asymmetric dimeric photosensitizers.

## 2. Materials and Methods

To develop photosensitizers with dual imaging and therapeutic functionalities guided by high-performance theoretical methods, it is essential to systematically investigate key parameters such as the geometric structures, electronic properties, adiabatic energies, and transition characteristics of different electronic states. In this study, Density Functional Theory (DFT) was employed to optimize the ground-state geometries and perform frequency analysis for BODIPY derivatives [17,18,19]. The 6-31+G(d,p) basis set was selected in combination with the M06-2X functional, which is well-suited for calculating thermochemistry, kinetics and electronic excitation energies of main-group elements [20]. To account for solvent effects, the SMD implicit solvation model was applied for the thieno-fused bis-BODIPY system in toluene solution. Vibrational frequency analysis confirmed that all optimized geometries correspond to true energy minima for both ground and excited states. All the calculations described above were carried out using the Gaussian 16 software package.

Furthermore, frontier molecular orbital (FMO) analysis, localized π-electron analysis, and electrostatic potential analysis were performed using the Multiwfn 3.8 and VMD 8.5.6 programs [21,22,23,24,25]. Spin–orbit coupling (SOC) matrix elements were computed with the PBE0 functional and the cc-PVDZ-DK basis set via the BDF 2022A software package. Finally, the MOMAP 2021B program was utilized to calculate the decay rate constants of the molecular excited states [26,27].

More important, for all molecules, the T_1_ and T_2_ states are nearly degenerate in energy, and the T_2_ state is unstable (it can rapidly convert to T_1_ via vibrational relaxation). Therefore, only the T_1_ state data are reported throughout the text.

## 3. Results and Discussion

### 3.1. Oxidation Potential of Electron-Donating Groups

To further verify the relative electron-donating abilities of methoxybenzene and pyrrole, the oxidation potentials of both compounds were calculated using ORCA 4.2.1 [28]. A smaller oxidation potential indicates a stronger electron-donating ability of the group [29]. The calculation formula is as follows Equations (1) and (2):

(1) $$\Delta G_{OX}^{O}=∆G_{gas}^{O}+∆G_{solv}^{mod}\left(A^{+}\right)-∆G_{solv}^{mod}(A)$$

(2) $$E_{OX}=∆G_{OX}^{O}-4.280$$

where $\Delta G_{OX}^{O}$ is the Gibbs free energy change; $∆G_{gas}^{O}$ is the gas-phase free energy change; $∆G_{solv}^{mod}\left(A^{+}\right)$ is the solvation free energy of the molecular cation; $∆G_{solv}^{mod}(A)$ is the solvation free energy of the molecule; and 4.280 V is the standard hydrogen electrode potential.

The results show that the oxidation potential of methoxybenzene is 2.700 V, while that of pyrrole is 2.615 V, indicating that the pyrrole group has a stronger electron-donating ability. This, in turn, facilitates intramolecular charge transfer and enhances the ISC process.

### 3.2. The Geometric Structure Differences in FD1–FD5

**Figure 2:**
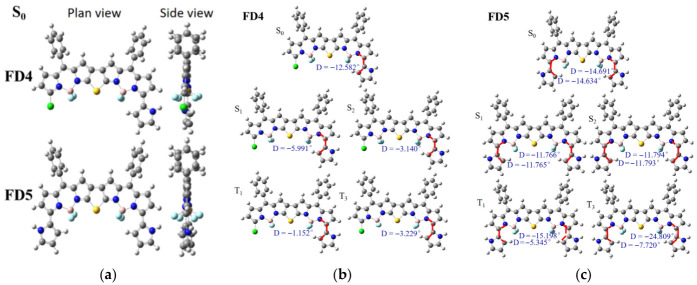
(**a**) Plane view and side view for FD4 and FD5 in S_0_ state; (**b**) plane view for FD4 in S_0_, S_1_, S_2_, T_1_ state; (**c**) plane view for FD5 in S_0_, S_1_, S_2_, T_1_ state.

Molecular structure fundamentally determines molecular properties. We first examined the skeletal framework and the spatial arrangement of the meso-phenyl groups. Table S1 lists the dihedral angles between the thiophene ring and the two adjacent BODIPY cores for compounds FD1–FD5 in the ground state (S_0_) and excited states (S_1_, S_2_, T_1_, and T_3_). As shown in Figure 2a (structures of FD1–FD3 are provided in Figure S1), the front and side views of the three-dimensional geometries of FD4 and FD5 in the ground state clearly demonstrate that the thiophene ring and the two flanking BODIPY units in FD1–FD5 are nearly coplanar. This high degree of in-plane coplanarity facilitates the formation and stabilization of an extended intramolecular π-conjugated system. In contrast, the meso-phenyl groups exhibit significant torsional distortion relative to the thieno-fused BODIPY plane, with the absolute values of the torsion angles consistently around 50.000° (Table S1). Moreover, this twisted conformation remains essentially unchanged across different electronic states, indicating considerable conformational stability. To gain deeper insight into the structure–property relationship between the α-substituents of BODIPY and the thieno-fused double-BODIPY core, we systematically analyzed the dihedral angle variations in FD1 (Figure S2), FD2 (Figure S3), FD3 (Figure S4), FD4 (Figure 2b), and FD5 (Figure 2c) across different electronic states. The results show that in the singlet excited states (S_1_, S_2_), the substituents in all molecules exhibit a planarization trend, whereas in the triplet states (T_1_, T_3_), higher structural asymmetry and distortion are generally observed. Specifically, the degree of planarization is jointly governed by the substitution pattern and the electronic nature of the substituent: for the same substituent type, monosubstitution (e.g., FD4) leads to significantly stronger planarization than disubstitution (e.g., FD5); under the same substitution pattern, the stronger electron-donating pyrrole group (FD4) induces much more pronounced planarization compared with the methoxybenzene group (FD2). These findings indicate that a stronger electron-donating group combined with a monosubstitution pattern most effectively promotes planarization of the molecular skeleton and structural relaxation in the excited states.

### 3.3. Molecular Orbital Characteristics of Thieno-Bis-BODIPY

**Figure 3:**
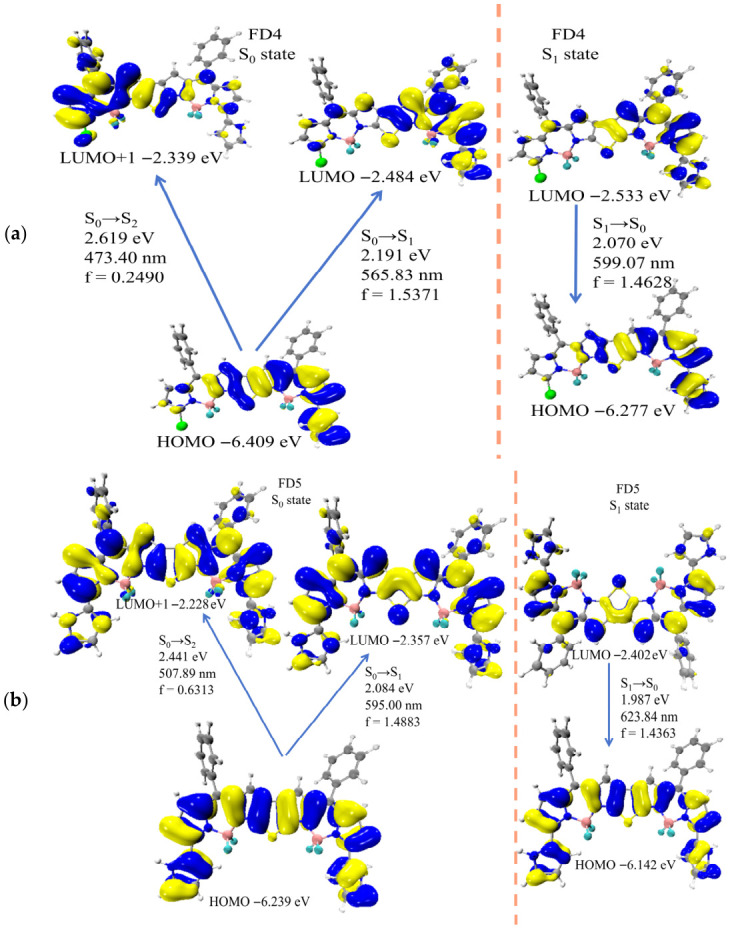
(**a**) Frontier molecular orbitals of FD4 in S_0_ state and S_1_ state; (**b**) Frontier molecular orbitals of FD5 in S_0_ state and S_1_ state.

For the absorption process, to systematically investigate the influence of structural differences on electronic transition properties, we compared the transition characteristics from the ground state (S_0_) to low-lying excited states (S_1_ and S_2_) for each molecule—specifically for FD1–FD3 (Figures S5–S7) and FD4–FD5 (Figure 3a,b). All molecules can undergo S_0_ → S_1_ transitions dominated by HOMO → LUMO, with relatively large oscillator strengths, indicating strong transition probability for FD1–FD5 along this pathway. In contrast, the S_0_ → S_2_ transitions (mainly contributed by HOMO → LUMO + 1) show clear structural dependence in oscillator strength: FD1 (Cl-substituted) exhibits the lowest value (0.0947). As the electron-donating ability of the substituent increases (from methoxybenzene to pyrrole), the oscillator strength gradually rises (FD3: 0.4946; FD5: 0.6313).

Moreover, the substitution pattern also significantly affects the transition intensity—disubstituted systems (FD3, FD5) display markedly higher oscillator strengths than their monosubstituted counterparts (FD2, FD4), suggesting that dual substitution enhances the allowedness of higher-energy transitions. Regarding the emission process, the radiative S_1_ → S_0_ transition in all molecules is dominated by LUMO → HOMO, with highly overlapping electron cloud distributions for this orbital pair. For FD1, this process yields an emission energy of 2.248 eV (551.57 nm) with an oscillator strength of 1.5295. The oscillator strengths for FD2–FD5 are 1.5292, 1.5006, 1.4628, and 1.4363, respectively, confirming the strong fluorescence capability of this thieno-fused double-BODIPY series (Figures S5–S7 and Figure 3a,b). From FD1 to FD5, the S1 → S0 emission energy decreases sequentially (2.248 eV → 2.137 eV → 2.072 eV → 2.070 eV → 1.987 eV), corresponding to a gradual red-shift in emission wavelength, which aligns with the experimentally observed fluorescence trend (659 nm, 698 nm, 713 nm, 718 nm, and 740 nm, respectively).

Through further localized LUMO-π molecular orbital analysis of FD1–FD5, the electronic structural characteristics of thieno-bis-BODIPYs were elucidated. As shown in Figure S8, the frontier molecular orbitals (FMOs) of FD1–FD5 exhibit significant delocalization of π-electron density across the entire molecular scaffold. This extensive π-conjugation pathway effectively narrows the HOMO–LUMO energy gap, thereby explaining the relatively red-shifted absorption and emission wavelengths observed in these derivatives [30,31].

In summary, the electronic nature of the substituent and its substitution pattern cooperatively modulate the photophysical properties of the system. Specifically, the introduction of stronger electron-donating groups at the α-position of the BODIPY units induces a pronounced red-shift in both the absorption and emission wavelengths of the Thieno-bridged bis-BODIPY system. Moreover, while a monosubstitution pattern promotes structural planarity and facilitates the ISC process, a bisubstitution pattern leads to greater structural distortion and a further bathochromic shift, which conversely favors fluorescence emission. Consequently, this enhanced fluorescence channel competes with and effectively suppresses the ISC pathway.

### 3.4. Electron Excitation Characterization of Thieno-Bis-BODIPY

**Figure 4:**
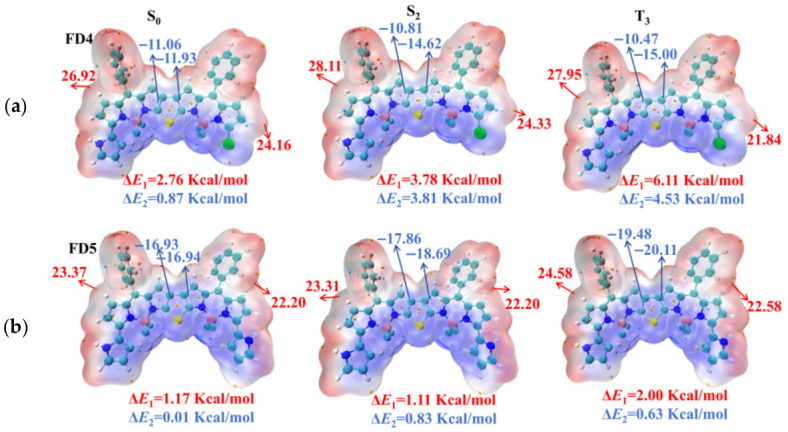
(**a**) The surface electrostatic potential of FD4 and (**b**) FD5 in S_0_ state, S_2_ state and T_3_ state.

To further investigate the intramolecular charge distribution, we analyzed the surface ESP at critical junctions. The greater electrostatic potential difference promotes exciton dissociation, which in turn drives more pronounced electron redistribution and transfer, thereby increasing the proportion of CT states. These findings provide key insights into understanding the excited-state electronic behavior of these molecules [32,33]. Specifically, ΔE_1_ (marked in red) represents the surface electrostatic potential difference at the junction between the α-substituents on both sides and the BODIPY core, while ΔE_2_ (marked in blue) denotes the corresponding difference at the connection between the substituted BODIPY unit and the central thiophene ring. By analyzing the surface electrostatic potential (ESP) of FD1–FD5 under different electronic states (FD1: Figure S9; FD2, FD3: Figure S10; FD4: Figure 4a; FD5: Figure 4b), it is found that the monosubstituted molecules (FD2, FD4) consistently exhibit significantly larger ESP differences at both the α-position junctions (ΔE_1_) and the thiophene ring regions (ΔE_2_) compared to their corresponding disubstituted counterparts (FD1, FD3, FD5), with these differences further increasing in excited states (S_2_, T_3_). Specifically, in the T_3_ state, the pyrrole-monosubstituted FD4 shows the largest ΔE_1_ (6.11 kcal·mol^−1^) and ΔE_2_ (4.53 kcal·mol^−1^), which are higher than those of the disubstituted pyrrole derivative FD5 (2.00 and 0.63 kcal·mol^−1^) as well as the methoxybenzene-monosubstituted FD2 (4.49 and 3.00 kcal·mol^−1^). This indicates that the asymmetric substitution pattern, together with stronger electron-donating ability, synergistically enhances the electrostatic potential gradient at key molecular sites, thereby increasing the CT proportion of the S_2_ and T_3_ states of FD4.

**Figure 5:**
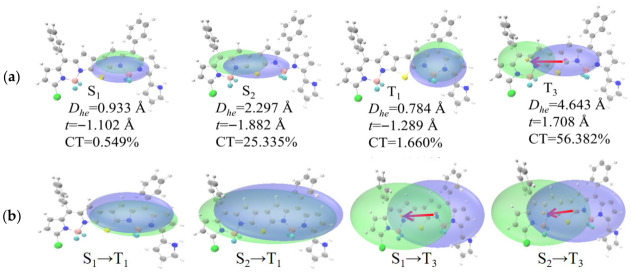
(**a**) Electron and hole map of FD4 in the S_1_, S_2_, T_1_, and T_3_ states; (**b**) Electron density difference diagram of FD4 during S_1_ → T_1_, S_2_ → T_1_, S_1_ → T_3_ and S_2_ → T_3_ transitions. Isovalue = 0.0003.

**Figure 6:**
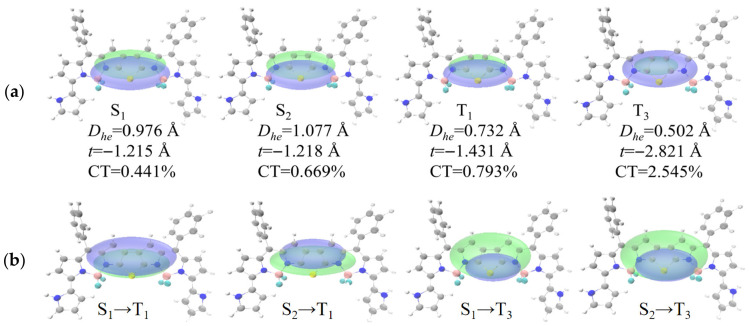
(**a**) Electron and hole map of FD5 in the S_1_, S_2_, T_1_, and T_3_ states; (**b**) Electron density difference diagram of FD5 during S_1_ → T_1_, S_2_ → T_1_, S_1_ → T_3_ and S_2_ → T_3_ transitions. Isovalue = 0.0003.

To investigate the ISC mechanism of thieno-fused double-BODIPY molecules, we systematically analyzed the electronic behavior of excited states by combining electron-hole maps, electron density difference maps (FD1–FD3: Figures S11–S13; FD4: Figure 5; FD5: Figure 6 and net charge-transfer heatmaps (Figures S14 and S15). The characteristics of excited states can be described using the distance between hole and electron centroids (D_he_) and the proportion of charge transfer (CT) contribution: a smaller D_he_ value corresponds to a lower CT contribution (indicating dominant localized excitation), while a larger D_he_ value corresponds to a significant CT contribution, reflecting charge-transfer characteristics [34]. Analysis shows that the S_1_ states of all studied molecules (FD1–FD5) exhibit typical local excitation (LE) characteristics (D_he_ ≤ 2.297 Å; CT ≤ 1%), which limits the S_1_ → T_n_ ISC process. Meanwhile, in the (FD1) and the disubstituted systems (FD3, FD5), the S_2_ state also shows typical LE features (FD1: D_he_ = 0.481 Å, CT = 8.379%; FD3: D_he_ = 0.975 Å, CT = 0.784%; FD5: D_he_ = 1.077 Å, CT = 0.669%), and the T_1_ and T_3_ states likewise retain highly localized characteristics (D_he_ ≤ 1.494 Å, CT ≤ 3%), thereby restricting the S_2_ → T_n_ ISC process. In contrast, the monosubstituted systems (FD2, FD4) exhibit significantly different excited-state behavior: their S_2_ states (FD2: D_he_ = 1.278 Å, CT = 12.547%; FD4: D_he_ = 2.297 Å, CT = 25.335%) show mixed LE/CT character. Although the T_1_ state remains predominantly LE (CT contributions of 1.852% and 1.660%, respectively), the T_3_ state displays pronounced charge-transfer character, with D_he_ values around 4.351–4.643 Å, significantly increased CT contributions (FD2: 52.676%; FD4: 56.382%), and substantial net charge transfer (FD2: 0.527 e; FD4: 0.564 e), thus favoring the S_2_ → T_n_ ISC pathway. Electron density difference maps further reveal a pronounced electron redistribution during the S_1_/S_2_ → T_3_ transition, where electrons migrate from the substituted donor BODIPY unit to the opposite BODIPY region. Notably, the pyrrole-substituted FD4 exhibits stronger charge-separation character in the T_3_ state than the methoxy-substituted FD2, as reflected by its larger D_he_ value, higher CT contribution percentage, and greater net charge transfer, consistent with its stronger electron-donating capability. The transition from mixed LE/CT character in the S_2_ state to a predominantly LE T_1_ state or a pure CT T_3_ state enhances intersystem spin–orbit coupling, effectively promotes the ISC process, and provides clear electronic structure evidence for understanding how monosubstitution with strong electron-donating groups improves photosensitization efficiency.

In summary, the substitution pattern is a key factor in modulating excited-state properties: symmetric disubstituted systems retain LE character in all excited states, whereas asymmetric monosubstituted systems exhibit mixed LE/CT character in the S_2_ state, maintain LE character in the T_1_ state, and induce CT character in the T_3_ state, thereby forming a CT → LE/CT ISC pathway [35,36]. The stronger the donor (e.g., pyrrole), the more pronounced the charge separation, which provides favorable conditions for FD4 to achieve more efficient ISC compared to FD2.

### 3.5. The Thieno-Bi-Constitution Photodynamic Mechanism and Rate Constant

**Figure 7:**
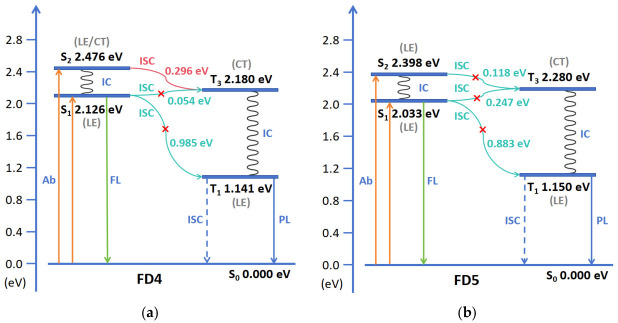
Jablonski energy level diagram of (**a**) FD4 and (**b**) FD5.

According to the data in Table 1, Figure 7 and Figures S16–S18, the adiabatic energy gaps (ΔE_ST_) between the S_2_ and T_1_ states of FD1–FD5 range from 0.835 to 0.985 eV. The large energy gap, combined with the highly LE character of the S_1_ state, hinders the S_1_ → T_1_ ISC process. Similarly, the ΔE_ST_ between S_2_ and T_1_ ranges from 1.217 to 1.335 eV, and the large gap together with the LE character of the S_2_ state (in disubstituted systems) also precludes S_2_ → T_1_ ISC. In contrast, the energy gaps from S_1_ and S_2_ to the T_3_ state are significantly smaller, providing favorable energetic conditions for ISC. In the disubstituted systems (FD3, FD5), although the S_1_ → T_3_ and S_2_ → T_3_ channels exhibit relatively narrow ΔE_ST_ values (0.118–0.247 eV) and appreciable spin–orbit coupling (SOC) values (0.07–0.42 cm^−1^), the highly LE nature of the S_1_ and S_2_ states suppresses ISC. In the monosubstituted systems (FD2, FD4), the S_1_ → T_3_ ΔE_ST_ is an order of magnitude smaller (0.032–0.054 eV), with corresponding SOC values of 0.20 and 0.14 cm^−1^. Although the energy levels and SOC are favorable for ISC, the LE character of the S_1_ state still limits this pathway. However, for the S_2_ → T_3_ channel in FD2 and FD4, the S_2_ state exhibits mixed LE/CT character (CT contributions: 12.547% for FD2, 25.335% for FD4), and the T_3_ state shows pronounced CT character, while the SOC values for this channel are significantly enhanced (0.46 and 0.49 cm^−1^, respectively), making it an efficient ISC pathway. Furthermore, the energy gaps between the T_1_ state and the ground state S_0_ for all molecules exceed 0.980 eV (actual range: 1.141–1.430 eV), indicating that their triplet-state energies are sufficient to sensitize triplet oxygen (^3^O_2_) to generate singlet oxygen (^1^O_2_). These results demonstrate that the sulfur-fused dimeric systems (FD2 and FD4) not only achieve efficient ISC via the S_2_ → T_3_ pathway but also show potential for application in photodynamic tumor therapy.

Further calculations were performed for the radiative decay rate constants (k_R_) and Sₙ-Tₙ intersystem crossing (ISC) rate constants of FD1–FD5 (Table 2). The results show that the k_R_ values of all five molecules are on the order of 10^8^ s^−1^, indicating that all of them possess fluorescence emission capability. Among them, the disubstituted systems (FD3 and FD5) exhibit higher k_R_ values than the monosubstituted systems (FD2 and FD4), suggesting that disubstitution is more favorable for fluorescence generation. Regarding the ISC process, the total k_ISC_ values of the monosubstituted molecules FD2 (1.336 × 10^7^ s^−1^) and FD4 (2.771 × 10^7^ s^−1^) are significantly higher than those of the disubstituted systems, indicating that the monosubstitution pattern greatly enhances ISC efficiency. Analysis of individual ISC channels reveals that although the rate constant for the S_1_ → T_3_ channel can reach the order of 10^6^ s^−1^, this channel is actually highly forbidden due to the strong LE character of the S_1_ state. Only the S_2_ → T_3_ channel plays a key role. In this channel, the k_ISC_ values of FD2 and FD4 reach 3.342 × 10^6^ s^−1^ and 2.387 × 10^7^ s^−1^, respectively, which are an order of magnitude higher than those of the other molecules (≤2.968 × 10^5^ s^−1^), demonstrating that S_2_ → T_3_ is the primary pathway responsible for the efficient ISC of FD2 and FD4. These results provide further kinetic evidence that monosubstituted systems, especially FD4 under the influence of the strong electron-donating ability of the pyrrole group, exhibit an excellent S_2_ → T_3_ ISC performance.

In summary, in monosubstituted systems, the strong electron-donating group (pyrrole) plays a decisive role in the photophysical properties of the system. Compared to the methoxy-substituted FD2, the pyrrole-substituted FD4 exhibits significantly enhanced electron-donating ability. This not only results in more pronounced charge-transfer character in its T_3_ state but also increases the electronic character difference between the T_3_ state and the locally excited S_1_/S_2_ states. Consequently, the interstate spin–orbit coupling is enhanced, effectively promoting the ISC channels dominated by S_1_ → T_3_ and S_2_ → T_3_. This process follows the SOCT-ISC rules, and the relatively small adiabatic energy gaps further improve the ISC efficiency [37]. Therefore, monosubstituted photosensitizers are predicted to possess the ability to generate ^1^O_2_ while retaining fluorescence imaging capability. Taking the better-performing FD4 as an example, the mechanism of ROS generation is as follows: after the photosensitizer molecule in the ground state (S_0_) is excited to the S_2_ state by light of a specific wavelength, it primarily reaches the T_3_ state via S_2_ → T_3_ intersystem crossing, then relaxes to the T_1_ state through IC, and finally generates ^1^O_2_ via energy transfer to molecular oxygen. The above theoretical predictions provide a foundation for subsequent experimental studies, and some conclusions still require experimental validation.

## 4. Conclusions

In thieno-bridged bis-BODIPY systems, the strength of the electron-donating group and the substitution pattern jointly determine their photophysical properties. Studies show that, compared to disubstitution, monosubstitution (e.g., FD2/FD4) leads to a more planar molecular geometry, generating a larger surface electrostatic potential difference (ΔE). This further induces hybrid LE/CT characteristics in the S_2_/T_3_ state, effectively promoting electronic rearrangement in the excited state and resulting in an intersystem crossing rate constant (k_ISC_) that is an order of magnitude higher than that of disubstituted systems. Among them, the stronger electron-donating pyrrole group (compared to methoxy) further lowers the oxidation potential. In FD4, this contributes to a smaller torsional angle, a higher ΔE, and a greater degree of charge transfer, thereby achieving optimal ISC efficiency. In contrast, although disubstitution causes a further red-shift in absorption and emission wavelengths due to structural distortion, it tends to enhance fluorescence emission, which competitively suppresses the ISC process. In summary, monosubstitution combined with a strong electron-donating group (exemplified by the pyrrole-substituted FD4) most effectively enhances ISC efficiency. By modulating the electrostatic potential difference and electron redistribution, it is expected to possess the strongest singlet oxygen generation capability while maintaining fluorescence emission. This work provides a clear molecular design strategy for developing novel BODIPY-based photosensitizers through a π-conjugation extension approach.

## Figures and Tables

**Table 1:** The adiabatic energy gap and SOC of thieno-fused bis-BODIPY molecules.

Value	FD1	FD2	FD3	FD4	FD5
S_0_-T_1_/[eV]	1.430	1.303	1.299	1.141	1.150
S_1_-T_1_/[eV]	0.897	0.901	0.835	0.985	0.883
S_2_-T_1_/[eV]	1.291	1.277	1.217	1.335	1.248
S_1_-T_3_/[eV]	0.040	0.032	0.198	0.054	0.247
S_2_-T_3_/[eV]	0.353	0.344	0.184	0.296	0.118
SOC_S1-T1_/[cm^−1^]	0.07	0.26	0.11	0.22	0.10
SOC_S2-T1_/[cm^−1^]	0.83	0.28	0.11	0.15	0.07
SOC_S1-T3_/[cm^−1^]	0.45	0.20	0.42	0.14	0.32
SOC_S2-T3_/[cm^−1^]	0.03	0.46	0.04	0.49	0.02

**Table 2:** Decay rate constants of excited state for thieno-fused bisBODIPY molecules.

Compound	*k*_R_/[s^−1^]	*k*_ISC_^1^/[s^−1^]	*k*_ISC(S1-T1)_/[s^−1^]	*k*_ISC(S2-T1)_/[s^−1^]	*k*_ISC(S1-T3)_/[s^−1^]	*k*_ISC(S2-T3)_/[s^−1^]
FD1	2.282 × 10^8^	4.193 × 10^6^	3.322 × 10^4^	2.301 × 10^4^	3.930 × 10^6^	2.071 × 10^5^
FD2	1.915 × 10^8^	1.336 × 10^7^	1.415 × 10^6^	8.925 × 10^5^	7.711 × 10^6^	3.342 × 10^6^
FD3	2.615 × 10^8^	4.838 × 10^6^	7.667 × 10^5^	1.894 × 10^5^	3.585 × 10^6^	2.968 × 10^5^
FD4	1.667 × 10^8^	2.771 × 10^7^	7.473 × 10^5^	2.793 × 10^5^	2.813 × 10^6^	2.387 × 10^7^
FD5	1.929 × 10^8^	7.291 × 10^6^	6.265 × 10^5^	1.522 × 10^5^	6.498 × 10^6^	1.431 × 10^4^

Note: k_ISC_ refers to the sum of the ISC rate constants of the molecule consisting of S_1_ → T_1_, S_2_ → T_1_, S_1_ → T_3_ and S_2_ → T_3_.

## Data Availability

The original contributions presented in this study are included in the article/Supplementary Materials. Further inquiries can be directed to the corresponding author.

## Supplementary Materials

The following supporting information can be downloaded at: https://www.mdpi.com/article/10.3390/ma19101987/s1, Figure S1: Plan view and side view of FD1–FD3; Figure S2: The dihedral angles of FD1 in the ground and electronic excited states; Figure S3: The dihedral angles of FD2 in the ground and electronic excited states; Figure S4: The dihedral angles of FD3 in the ground and electronic excited states; Figure S5: Frontier molecular orbitals of FD1 in S_0_ state and S_1_ state; Figure S6: Frontier molecular orbitals of FD2 in S_0_ state and S_1_ state; Figure S7: Frontier molecular orbitals of FD3 in S_0_ state and S_1_ state; Figure S8: LMO-π electron coloring plot under thieno-fused bisBODIPY molecules 1.000 Å; Figure S9: The surface electrostatic potential of FD1 in S_0_ state, S_2_ state and T_3_ state; Figure S10: The surface electrostatic potential of FD2 and FD3 in S_0_ state, S_2_ state and T_3_ state; Figure S11: (a) Electron and hole map of FD1 in the S_1_, S_2_, T_1_, and T_3_ states; (b) Electron density difference diagram of FD4 during S_1_ → T_1_, S_2_ → T_1_, S_1_ → T_3_ and S_2_ → T_3_ transitions. Isovalue = 0.0003; Figure S12: (a) Electron and hole map of FD2 in the S_1_, S_2_, T_1_, and T_3_ states; (b) Electron density difference diagram of FD4 during S_1_ → T_1_, S_2_ → T_1_, S_1_ → T_3_ and S_2_ → T_3_ transitions. Isovalue = 0.0003; Figure S13: (a) Electron and hole map of FD3 in the S_1_, S_2_, T_1_, and T_3_ states; (b) Electron density difference diagram of FD4 during S_1_ → T_1_, S_2_ → T_1_, S_1_ → T_3_ and S_2_ → T_3_ transitions. Isovalue = 0.0003; Figure S14: Heat diagram of thieno-fused bisBODIPY molecule in S_2_ state; Figure S15: Heat diagram of thieno-fused bisBODIPY molecule in T_3_ state; Figure S16: Jablonski energy level diagram of FD1; Figure S17: Jablonski energy level diagram of FD2; Figure S18: Jablonski energy level diagram of FD3; Table S1: The dihedrals of the thieno-fused bisBODIPY molecules in the ground state and electronic excited states.
