# Supplementary material for: Mechanism Research on Thieno-Fused Bis-BODIPY Bifunctional Photosensitizers
Source: Materials (Basel). 2026 May 11;19(10):1987. doi: 10.3390/ma19101987 (PMC13208951; doi:10.3390/ma19101987)
Supplement: Supplementary file 1 [file materials-19-01987-s001.zip › materials-4252348-supplementary.pdf]

materials

v

Electronic Supporting Information

Mechanism Research on Thieno-Fused Bis-BODIPY Bifunctional Photosensitizers

Yuejia Wang [1,2,†], Di Wang [1,†], Xinyu Chen [1], Yishan Sun [1,2], Guoguo Shi [1,2] and Jianfang Cao [1,*]

1 School of Chemical Engineering, Ocean and Life Sciences, Panjin Campus, Dalian University of Technology, Panjin 124221, China; wangdi0072@mail.dlut.edu.cn (D.W.); henxinyyy@163.com (X.C.)
2 Leicester International Institute, Panjin Campus, Dalian University of Technology, Panjin 124221, China; 2339120564@mail.dlut.edu.cn (Y.W.); bpjlbxx@mail.dlut.edu.cn (Y.S.); sgg1103@mail.dlut.edu.cn (G.S.)
* Correspondence: caojf@dlut.edu.cn
† These authors contributed equally to this work.

Content:

1. Geometric Structure 2
2. Orbital Characteristics 4
3. Electronic Excitation Characteristics 5
4. Jablonski energy level diagrams 8

1. Geometric Structure

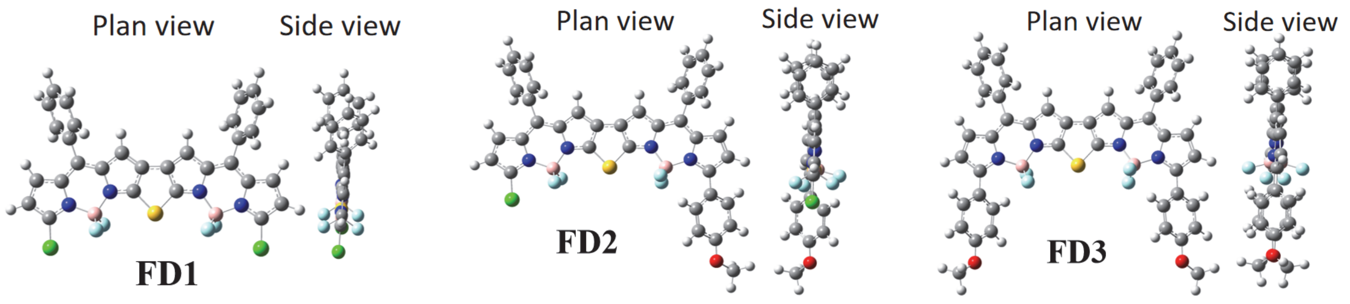

(a) (b) (c)

Figure S1. Plan view and side view of FD1-FD3.

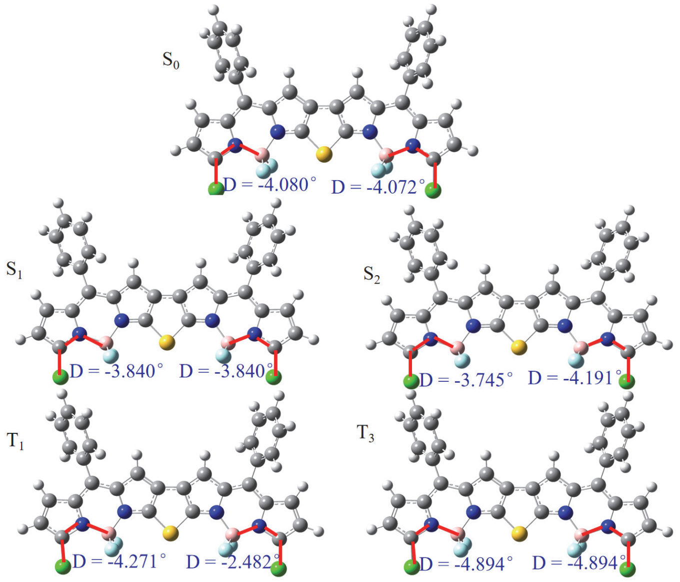

Figure S2. The dihedral angles of FD1 in the ground and electronic excited states.

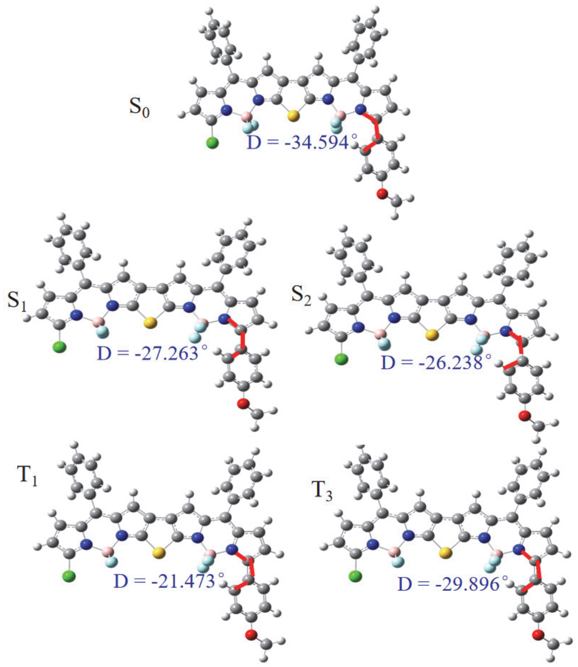

Figure S3. The dihedral angles of FD2 in the ground and electronic excited states.

Table S1. The dihedrals of the thieno-fused bisBODIPY molecules in the ground state and electronic excited states.

**Compound**	Dihedral°	C5-C6-C26-C33	C17-C22-C27-C32	N4-C10-S13-C14	C11-C15-C18-C17
	S_0	-55.512	-55.524	179.600	179.560
	S_1	-53.432	-53.431	179.662	179.700
FD1	S_2	-52.972	-52.972	179.256	179.126
	T_1	-55.317	-56.710	179.694	179.539
	T_3	-53.282	-55.818	179.771	179.688
	S_0	-55.296	-54.255	179.520	179.416
	S_1	-54.256	-50.664	179.708	179.290
FD2	S_2	-50.344	-53.549	178.781	179.849
	T_1	-55.190	-52.347	179.475	179.669
	T_3	-52.548	-54.955	179.451	179.867
	S_0	-54.321	-54.320	179.204	179.680
	S_1	-52.061	-52.063	179.051	179.622
FD3	S_2	-50.944	-50.942	178.819	179.353
	T_1	-54.160	-52.722	179.233	179.735
	T_3	-50.180	-54.820	178.054	179.908
	S_0	-55.033	-55.260	179.501	179.700
	S_1	-54.266	-52.581	179.627	179.546
FD4	S_2	-49.417	-55.223	178.786	179.889
	T_1	-54.982	-53.984	179.602	179.822
	T_3	-51.825	-55.993	179.299	-179.887
	S_0	-55.591	-55.590	179.459	179.709
	S_1	-53.712	-53.713	179.380	179.681
FD5	S_2	-52.261	-52.262	179.100	179.345
	T_1	-54.703	-55.489	179.250	179.819
	T_3	-56.909	-50.874	179.476	179.762

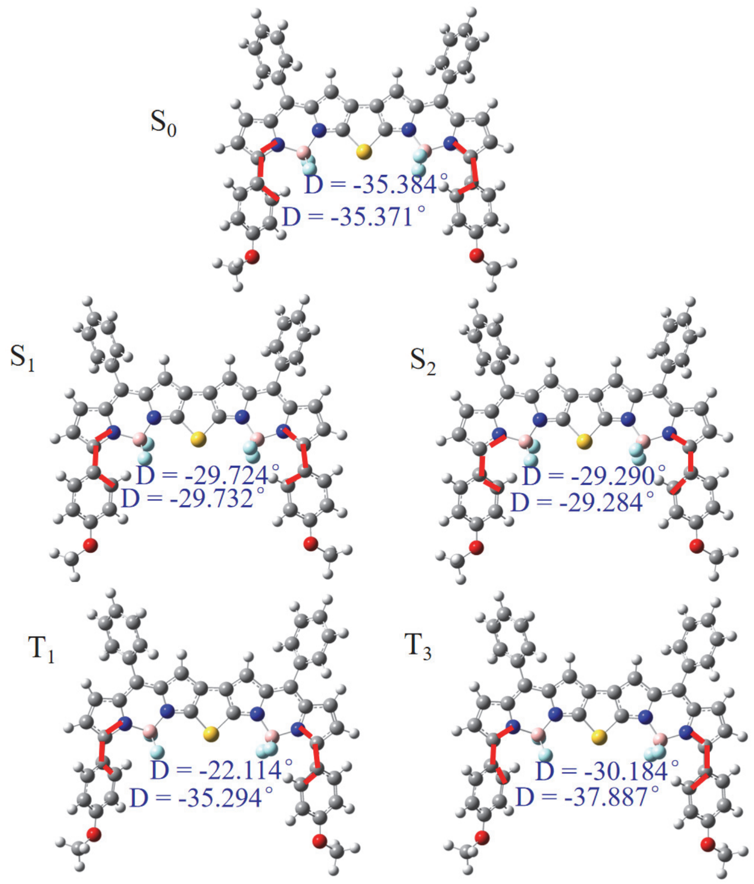

Figure S4. The dihedral angles of FD3 in the ground and electronic excited states.

2. Orbital Characteristics

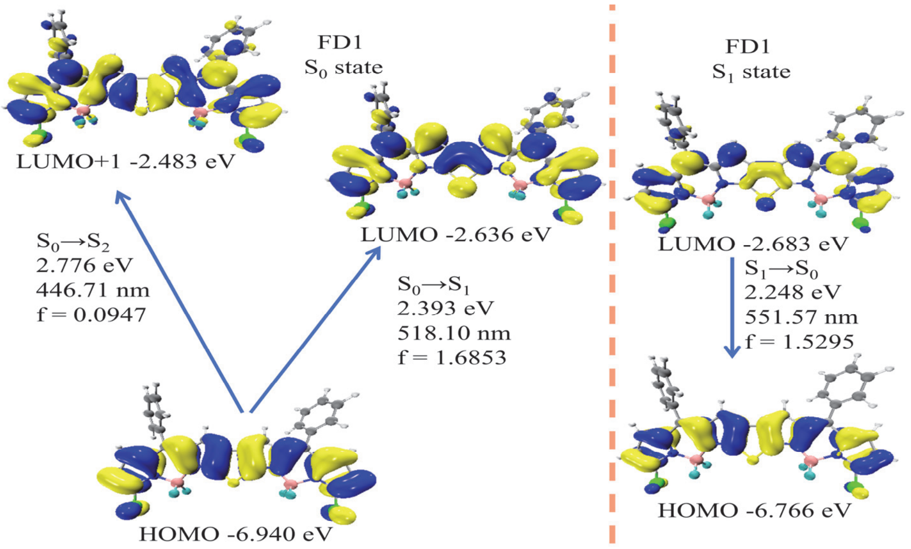

Figure S5. Frontier molecular orbitals of FD1 in S_0 state and S_1 state.

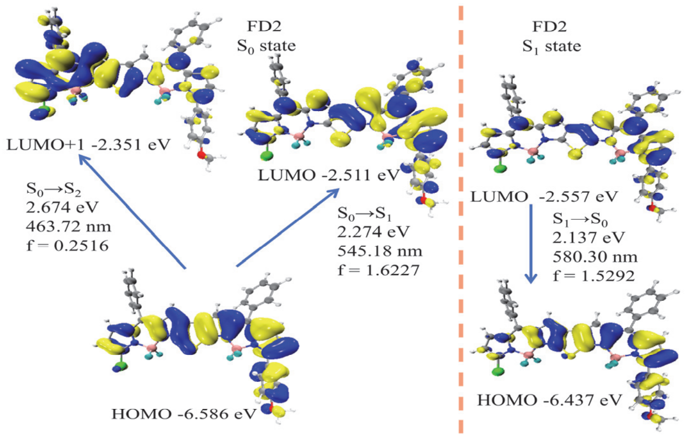

Figure S6. Frontier molecular orbitals of FD2 in S_0 state and S_1 state.

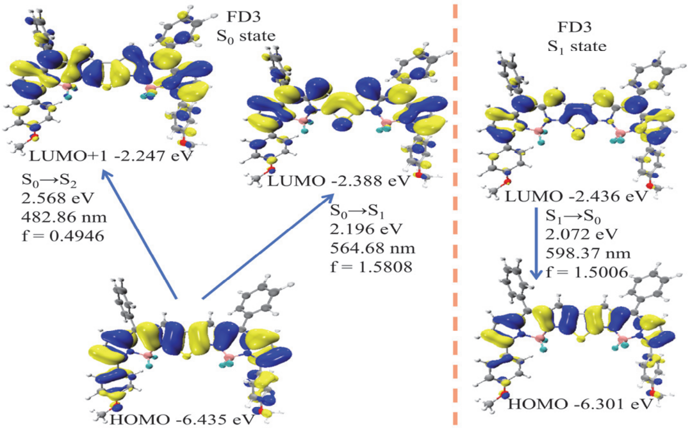

Figure S7. Frontier molecular orbitals of FD3 in S_0 state and S_1 state.

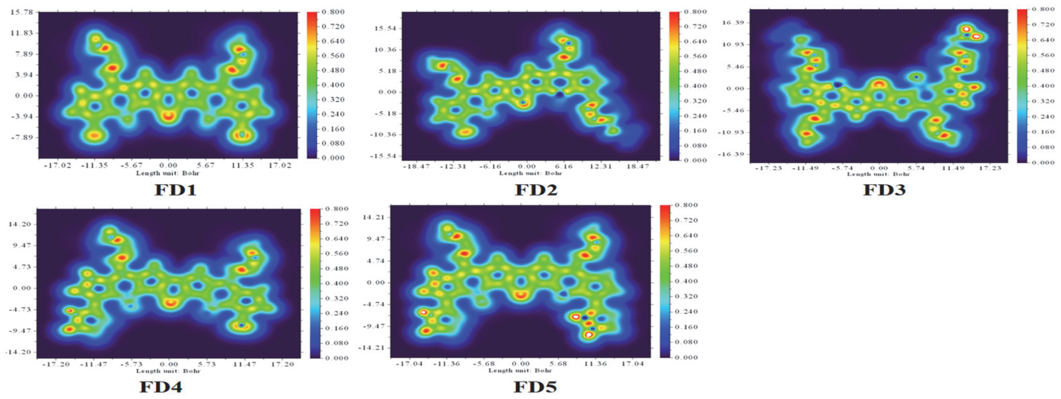

Figure S8. LMO-π electron coloring plot under thieno-fused bisBODIPY molecules 1.000 Å.

3. Electronic Excitation Characteristics

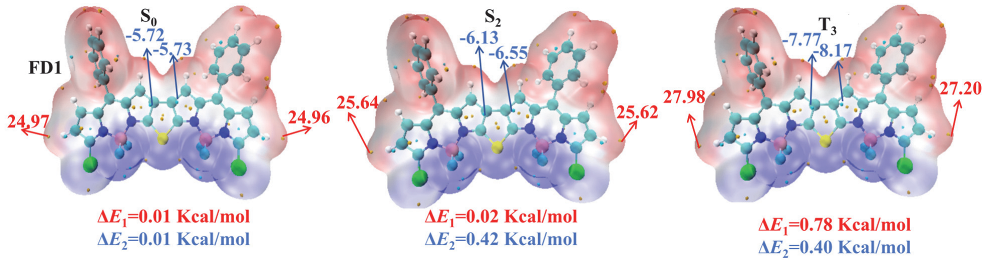

Figure S9. The surface electrostatic potential of FD1 in S_0 state, S_2 state and T_3 state.

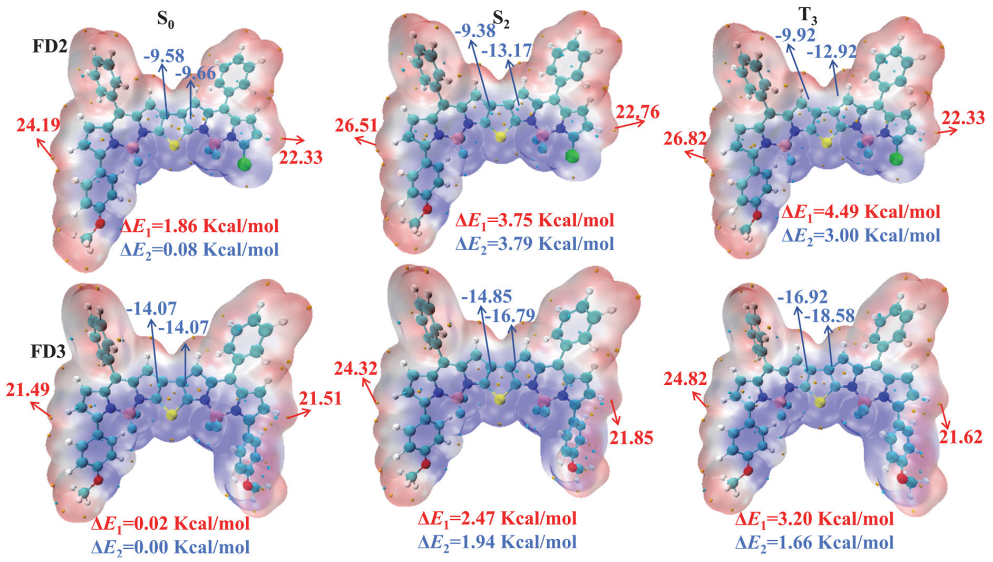

Figure S10. The surface electrostatic potential of FD2 and FD3 in S_0 state, S_2 state and T_3 state.

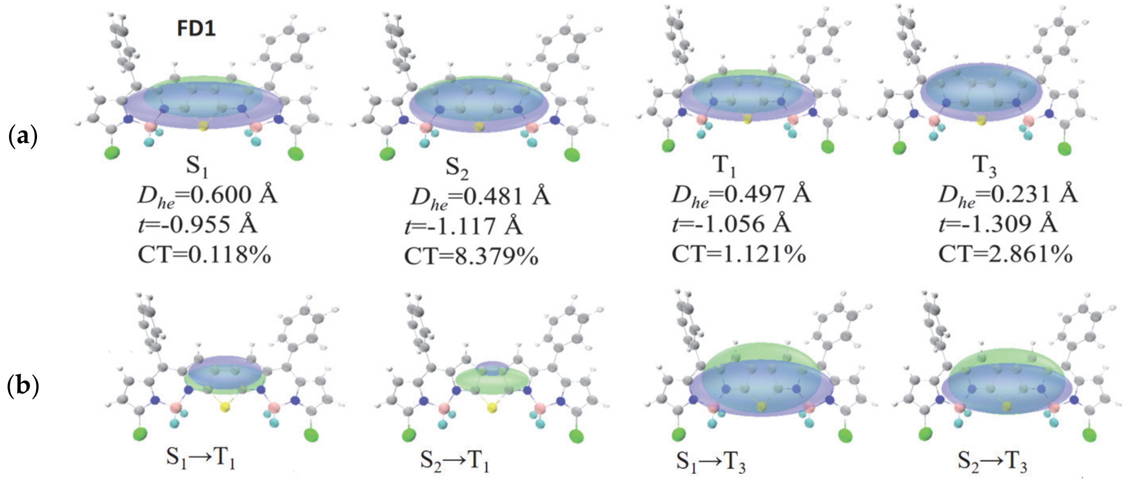

Figure S11. (a) Electron and hole map of FD1 in the S_1, S_2, T_1, and T_3 states; (b) Electron density difference diagram of FD4 during $S_1 \rightarrow T_1$, $S_2 \rightarrow T_1$, $S_1 \rightarrow T_3$ and $S_2 \rightarrow T_3$ transitions. Isovalue=0.0003.

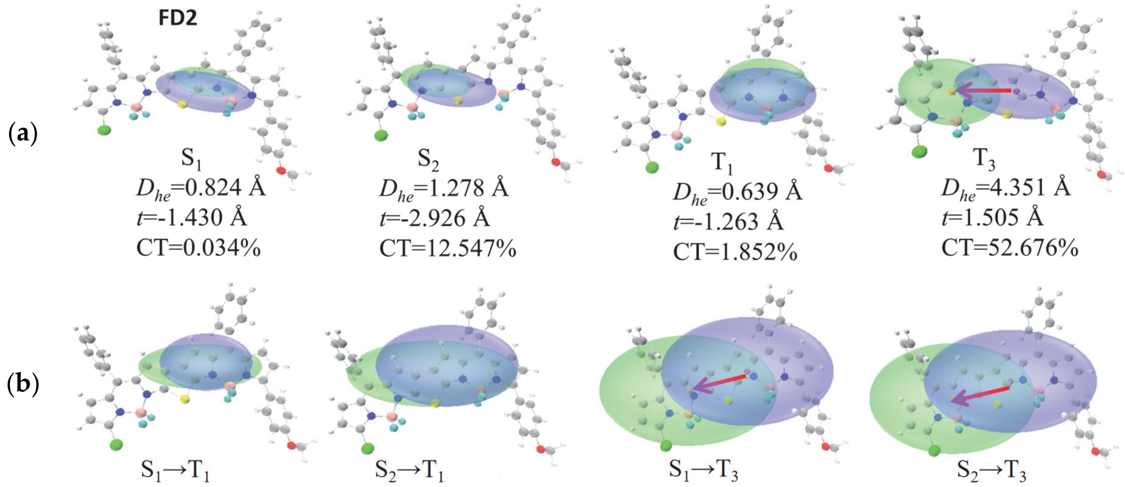

Figure S12. (a) Electron and hole map of FD2 in the S_1, S_2, T_1, and T_3 states; (b) Electron density difference diagram of FD4 during $S_1 \rightarrow T_1$, $S_2 \rightarrow T_1$, $S_1 \rightarrow T_3$ and $S_2 \rightarrow T_3$ transitions. Isovalue=0.0003.

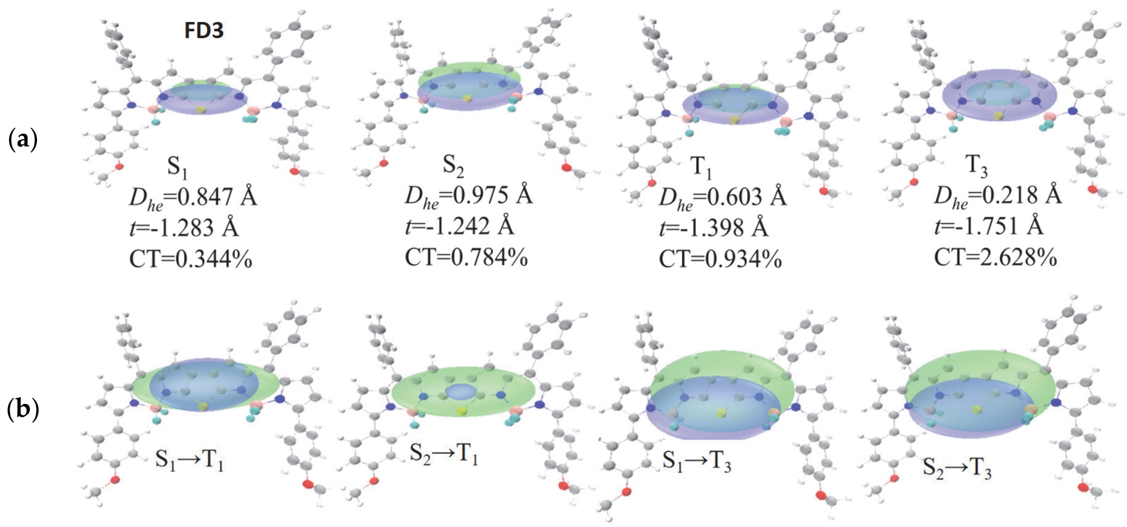

Figure S13. (a) Electron and hole map of FD3 in the S_1, S_2, T_1, and T_3 states; (b) Electron density difference diagram of FD4 during $S_1 \rightarrow T_1$, $S_2 \rightarrow T_1$, $S_1 \rightarrow T_3$ and $S_2 \rightarrow T_3$ transitions. Isovalue=0.0003.

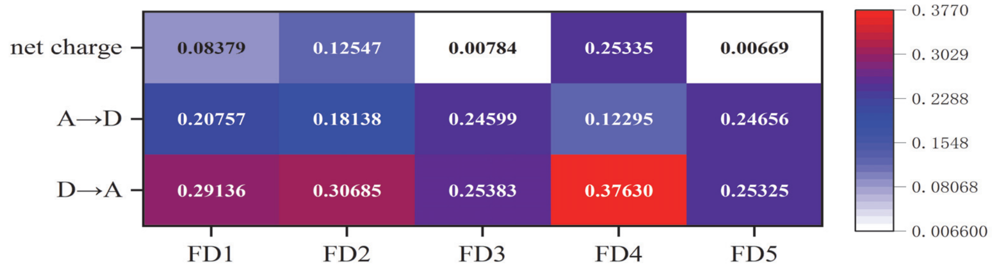

Figure S14. Heat diagram of thieno-fused bisBODIPY molecule in S_2 state.

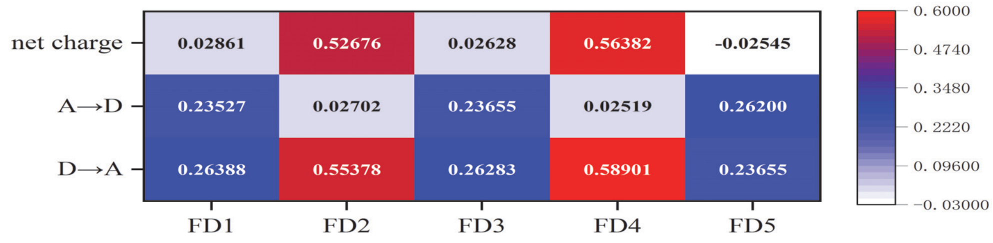

Figure S15. Heat diagram of thieno-fused bisBODIPY molecule in T_3 state.

4. Jablonski energy level diagrams

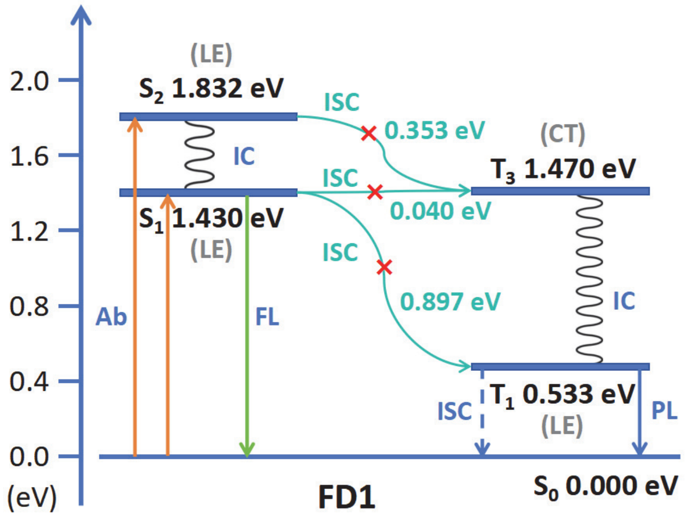

Figure S16. Jablonski energy level diagram of FD1.

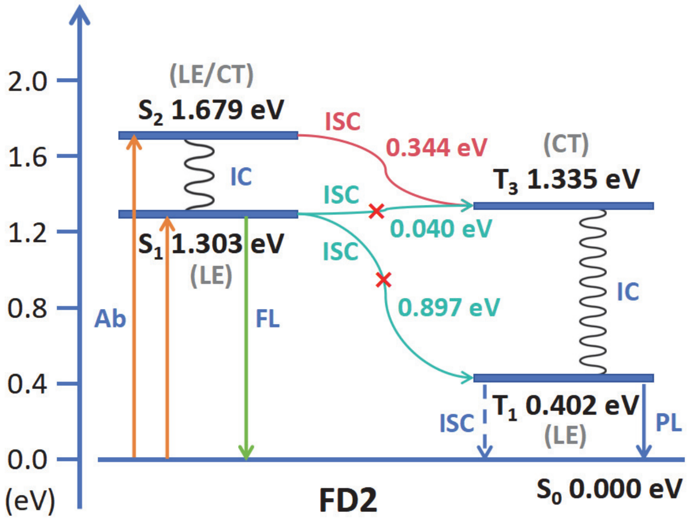

Figure S17. Jablonski energy level diagram of FD2.

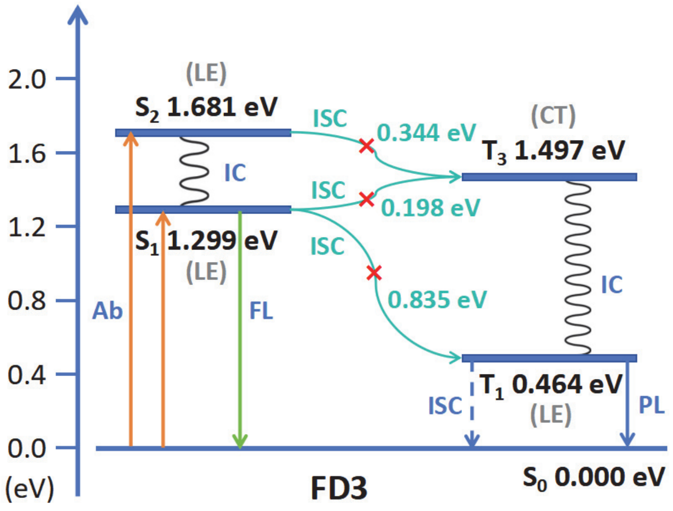

Figure S18. Jablonski energy level diagram of FD3.
